# Impact of Juglone, a PIN1 İnhibitor, on Oral Carcinogenesis Induced by 4-Nitroquinoline-1-Oxide (4NQO) in Rat Model

**DOI:** 10.3390/medicina60081192

**Published:** 2024-07-23

**Authors:** Olgun Topal, Burcu Güçyetmez Topal, Yunus Baş, Bünyamin Ongan, Gökhan Sadi, Esra Aslan, Betül Demirciler Yavaş, Mehmet Bilgehan Pektaş

**Affiliations:** 1 Department of Oral and Maxillofacial Surgery, Faculty of Dentistry, Afyonkarahisar Health Sciences University, 03200 Afyonkarahisar, Turkey; olgun.topal@afsu.edu.tr (O.T.); yunus.bas@afsu.edu.tr (Y.B.); b.ongan.1907@gmail.com (B.O.); 2 Department of Pedodontics, Faculty of Dentistry, Afyonkarahisar Health Sciences University, 03200 Afyonkarahisar, Turkey; burcu.topal@afsu.edu.tr; 3 Department of Biology, K.O. Science Faculty, Karamanoglu Mehmetbey University, 70100 Karaman, Turkey; sadigokhan@gmail.com; 4 Department of Histology and Embryology, Faculty of Medicine, Afyonkarahisar Health Sciences University, 03200 Afyonkarahisar, Turkey; dr_esragul@hotmail.com; 5 Private Practice, Traditional and Complementary Treatment Center, 03200 Afyonkarahisar, Turkey; betuldy@gmail.com; 6 Department of Medical Pharmacology, Faculty of Medicine, Afyonkarahisar Health Sciences University, 03200 Afyonkarahisar, Turkey

**Keywords:** oral cancer, carcinogenesis, juglone, apoptosis, Bax, Bcl-2

## Abstract

*Background and Objectives*: PIN1 is overexpressed in several human cancers, including prostate cancer, breast cancer, and oral squamous carcinomas. Juglone (J), derived from walnut, was reported to selectively inhibit PIN1 by modifying its sulfhydryl groups. In this study, the potential effects of juglone, also known as PIN1 inhibitor, on oral cancer and carcinogenesis were investigated at the molecular level. *Materials and Methods*: 4-Nitroquinoline N-oxide (4-NQO) was used to create an oral cancer model in animals. Wistar rats were divided into five groups: Control, NQO, Juglone, NQO+J, and NQO+J*. The control group received the basal diet and tap water throughout the experiment. The NQO group received 4-NQO for 8 weeks in drinking water only. The Juglone group was administered intraperitoneally in a juglone solution for 10 weeks (1 mg/kg/day). The NQO+J group received 4-NQO in drinking water for 8 weeks, starting 1 week after the cessation of 4-NQO treatment. They were then administered intraperitoneally in a juglone solution for 10 weeks. (1 mg/kg/day). NQO+J* group: received 4 NQO for 8 weeks in drinking water and administered intraperitoneally in a juglone solution for 10 weeks (1 mg/kg/day). They were sacrificed at the end of the 22-week experimental period. The tongue tissues of the rats were isolated after the experiment, morphological changes were investigated by histological examinations, and the molecular apoptotic process was investigated by rt-qPCR and western blot. *Results*: Histological results indicate that tumors are formed in the tongue tissue with 4-NQO, and juglone treatment largely corrects the epithelial changes that developed with 4-NQO. It has been determined that apoptotic factors p53, Bax, and caspases are induced by the effect of juglone, while antiapoptotic factors such as Bcl-2 are suppressed. However, it was observed that the positive effects were more pronounced in rats given juglone together with 4-NQO. *Conclusions*: The use of PIN1 inhibitors such as juglone in place of existing therapeutic approaches might be a promising and novel approach to the preservation and treatment of oral cancer and carcinogenesis. However, further research is required to investigate the practical application of such inhibitors.

## 1. Introduction

Oral cancer is the most common type of cancer in the head–neck region, and from the mid-2000s until recently (2015–2019), National Cancer Institute survey data have predicted that its incidence will increase in the coming years [1]. In particular, smoking and consuming alcohol are the main factors contributing to the risk of oral cancer [2]. In terms of clinical pathogenesis, poor oral hygiene; loose dentures; chronic irritation of teeth; nutritional deficiencies; ultraviolet light; radiation exposure; viral infections, including *Human papillomavirus* (HPV), *Epstein-barr* virus (EBV), and *Herpes simplex* virus (HSV); and fungal infections, including *Candida albicans*, are known to cause oral carcinogenesis [3]. Oral carcinogenesis involves a series of complex simultaneous stages that include the development of precancerous lesions and the processes of invasion and metastasis [4]. The main challenges in oral cancer can be listed as late diagnosis and epithelial-mesenchymal transition can promote resistance of cancer cells to a range of chemotherapeutic agents such as antimetabolites, platinum-based agents, and plant alkaloids which are FDA-approved [5,6]. Squamous cell carcinomas constitute 90% of oral cancers, and the five-year survival rate in this type has been reported to be approximately 56% [7]. It is known that many oral squamous cell carcinomas develop in association with a precancerous lesion, especially leukoplakia, or when these lesions undergo malignant transformation. It has been reported that it most commonly develops in the posterolateral or ventral tongue regions of the mouth. Other areas where it is seen are the floor of the mouth, soft palate, gingiva, cheek, lip, and hard palate [8]. The degree of the disease and the location of the tumor are important in the treatment planning of oral squamous cell carcinoma. Primary treatment is extensive surgical resection. Surgery may be followed by radiotherapy and/or chemotherapy depending on the pathological character of the tumor. While radiotherapy is not thought to be beneficial in early-stage carcinomas (stages Ι and ΙΙ) with negative surgical margins (stages ΙΙΙ and ΙV). Tumors with positive surgical margins or lymph node involvement with one or more extra-capsular spreads are considered to have a high risk of recurrence, and radiotherapy or chemo-radiation is recommended following surgery [9]. Chemotherapy can usually be applied together with radiotherapy, as initial treatment before chemo-radiation, or as palliative treatment. In addition, in the last 30 years, there have been treatment approaches in which only chemotherapy and radiotherapy are applied for “organ preservation” purposes, especially in larynx and oropharynx carcinomas. Survival rates of primary chemo-radiotherapy have been reported in a wide range of 29–66% [10]. Due to the potential side effects and morbidity risks of drugs used in the field of cancer, research on treatment with natural or herbal products constitutes an important source of studies in the development of chemotherapeutic strategies [11].

PIN1 is overexpressed in several human cancers, including prostate cancer, breast cancer, and oral squamous carcinomas. In cancer patients, a high expression of PIN1 correlates with a poor clinical outcome, lymph node metastasis in non-small-cell lung cancer patients, and disease progression in patients with oral squamous carcinoma. PIN1 overexpression induces chromosome instability and tumorigenesis. PIN1 inactivates and activates more than 26 tumor suppressors and 56 oncogenes, respectively [12]. PIN1 reduces the ΔNp63 ubiquitination induced by WWP1 to enhance the proliferation of oral squamous cell carcinoma [13].

In this context, recent studies point to the potential effects of phytotherapeutics that inhibit PIN1 in cancer treatment. Under stress, cells release pro-apoptotic proteins, such as Bax (Bcl-2-associated X protein) and caspases to initiate apoptosis under the control of cytosolic p53 (Tumor protein 53). These proteins also deactivate these structures by forming complexes with anti-apoptotic factors such as Bcl-xl (B-cell lymphoma xl) and Bcl-2 (B-cell lymphoma 2). This entire process is catalyzed by the PIN1 enzyme. Thus, apoptosis is controlled through conformational change of cytosolic p53 [14].

It is well established that numerous drugs employed in the treatment of cancer are derived from natural products and their derivatives. In this context, herbal products (phytochemicals) represent a significant source of research into the development of more effective cancer preventives and chemotherapeutic strategies. Juglone, an important herbal source, is a naturally occurring naphthoquinone derivative compound found in the roots, leaves, fruit pericarp, bark, and tree trunk of trees belonging to the Juglans (walnut) genus. It is widely used in Chinese, Indian, and Korean traditional medicine. The link between Pin1 and cancer was first suggested by studies showing the overexpression of Pin1 in cancer tissues. Juglone, derived from walnut, was reported to selectively inhibit Pin1 by modifying its sulfhydryl groups and the activities of other PPIase families is not affected by juglone. Juglone has been used in a number of studies depending on its function inhibiting Pin1 [15]. Recent studies point to the anti-inflammatory, cytotoxic, anti-tumor, antibacterial, and antioxidant effects of juglone [16,17,18]. Importantly, juglone, which is known to induce the apoptotic process through PIN1 inhibition, has been shown to have anti-tumor effects in pancreatic, prostate, melanoma, colorectal, glioblastoma, and breast cancers [19,20,21,22,23,24]. Although there are many studies on the tumor suppressor effects of juglone, its effects on oral cancer types remain unknown. Therefore, in this study, we aimed to investigate both the prophylactic and therapeutic effects of juglone by creating an oral cancer model with 4-NQO in rats.

## 2. Materials and Methods

### 2.1. Animals

The study protocol was approved by the Ethical Animal Research Committee of Afyon Kocatepe University (49533702/291) and it was designed in accordance with the ARRIVE guidelines/checklist.

The sample size was estimated using the G Power program (version 3.1.9.6). According to a previous study [25], the sample size estimation was performed based on a type I error (α) of 0.05, and power of the study at 95%, which indicated that 10 rats were required for each group (two-tailed hypothesis, effect size = 2.739). The sample size was increased to 12 rats for each group, allowing for loss due to incomplete examination.

Three-month-old, male, approximately 300 g, Wistar 60 rats were obtained from Afyon Kocatepe University Experimental Animal Application and Research Center. Standart rodent chow (Korkutelim Yem Sanayi A.Ş., Antalya/Türkiye) that composed of 62% starch, 23% protein, 4% fat, 7% cellulose, vitamins, and salt mixture were given ad libitum with drinking water during the experiment. Before practical applications, rats were acclimatized for one week, and then five groups were formed: Control, NQO, Juglone, NQO+J (those given juglone after creating an oral cancer model), and NQO+J* (those given juglone with oral cancer model). They were divided into groups of 12 rats each. The control group received the basal diet and tap water throughout the experiment and served as an untreated control. The NQO group received 4-NQO during 8 weeks in drinking water only. The Juglone group was administered intraperitoneally in a juglone solution for 10 weeks (1 mg/kg/day). The NQO+J group received 4-NQO in drinking water for 8 weeks, starting 1 week after the cessation of 4-NQO treatment. They were then administered intraperitoneally in a juglone solution for 10 weeks (1 mg/kg/day). NQO+J* group: received 4 NQO for 8 weeks in drinking water and administered intraperitoneally in a Juglone solution for 10 weeks (1 mg/kg/day).

### 2.2. Protocols

4-NQO (4-Nitroquinoline N-oxide) solution was prepared as 50 ppm (0.05 mg/mL) in drinking water. After body weighing the rats, we administered 4-NQO to the NQO, NQO+J, and NQO+J* groups in the drinking water of the rats for 8 weeks. Juglone was dissolved in 100% ethanol 14 mM (2.44 mg/mL stock solution). A dosage adjustment was made to achieve 1 mg/kg body weight in a final volume of 2 mL per injection with saline. Juglone injections were applied to the NQO+J group after being given 4-NQO for 8 weeks, and it was applied to the NQO+J* group together with 4-NQO application for 8 weeks. However, 2 mL saline injections were applied to the Control and NQO groups for 8 weeks. At the end of the experiment, the animals were anesthetized and decapitated with a combination of ketamine (100 mg/kg) and xylazine (10 mg/kg). Tongue tissue samples from the implant site were isolated from animals and stored at −85 °C for rt-PCR measurements; for histological analysis, it was stored in 10% formalin solution.

### 2.3. Determination of the Gene Expressions with rt-qPCR

Total RNA was isolated from 50 mg of tongue tissues with RNeasy total RNA isolation kit (Qiagen, Venlo, The Netherland) as described in the manufacturer protocol. After isolation, amount and the quality of total RNA were determined by spectrophotometry at 260/280 nm with ThermoScanGO microplate reader (Thermo Fisher Scientific, Waltham, MA, USA). Presence of any RNA degradation was checked for with agarose gel electrophoresis. cDNA synthesis was carried out by using 500 ng of total RNA and oligo (dT)18 primer using commercial first strand cDNA synthesis kit (Thermo Scientific, USA) as described by the supplier. Gene expressions of interested proteins were determined with real time PCR by mixing 1 μL cDNA, 5 μL SYBR Green Mastermix (Roche FastStart Universal, SYBR Green Master Mix) and primer pairs (Table 1) at 0.5 µM final concentrations each in a final volume of 10 μL. The real time PCR program of the quantitative PCR (LightCycler480 II, Roche, Basel, Switzerland) was arranged as follows: initial denaturation at 95 °C for 10 min, denaturation at 95 °C for 10 s, annealing at 58 °C for 15 s and extension at 72 °C for 15 s with 40 times repeated thermal cycle measuring the green fluorescence at the end of each extension steps. PCR reactions were carried out in triplicate, and the specificity of PCR products was determined through melt analysis. Additionally, negative controls lacking template were used in all reactions. Relative expressions of genes with respect to internal control GAPDH were calculated with the efficiency corrected advance relative quantification tool of the LightCycler^®^ 480 SW 1.5.1 software.

### 2.4. Determination of Protein Expressions by Western Blot

For the determination of p53, Bax, Bcl-2, Caspase-9, and Caspase-3 protein contents, tongue tissue was homogenized in 2-fold volumes of homogenization medium (50 mM Tris pH:7.4, 150 mM NaCl, 5 mM EDTA, 1% (*w*/*w*) Triton X-100, 0.26% (*w*/*v*) sodium deoxycholate, 50 mM sodium fluoride, 0.1 mM sodium orthovanadate, and 0.2 mM phenylmethylsulfonyl fluoride (PMSF)) with a Tissue RuptorTM (Qiagen, Netherlands) homogenizer. The homogenates were centrifuged at 1500× *g* for 10 min at 4 °C. After the removal of the supernatants, the protein concentrations were determined using the Lowry method [26]. Then, 50–100 micrograms of total proteins were separated using SDS-PAGE and transferred onto PVDF membranes using a semi-dry electroblotting apparatus (TransBlot Turbo, BioRad, Feldkirchen, Germany). Blotted membranes were then blocked with 5% (*w*/*v*) nonfat dried milk and incubated with primary antibodies for p53 (Anti-TP53 antibody, 1/1000), Bax (Anti-Bax antibody, 30–110 Internal, 1/1000), Bcl-2 (Anti-Bcl-2 antibody, 40–120, 1/1000), caspase-9 (Anti-CASP9 antibody, 60–140, 1/1000), and caspase-3 (Anti-CASP3 antibody, 1/1000) for 2 h at room temperature or overnight at 4 °C. As an internal control, GAPDH proteins were also labeled with anti-GAPDH Rabbit IgG (Santa Cruz, sc:25778, 1:2000). Horseradish-peroxidase-conjugated secondary antibody (Santa Cruz, sc:2030 or sc:2770, 1:10,000) was incubated for 1 h, and the blots were incubated in Clarity^TM^ Western ECL (Bio-Rad Laboratories, Hercules, CA, USA) substrate solution. Images of the blots were obtained using the ChemiDoc^TM^ MP Chemiluminescence detection system (Bio-Rad Laboratories, Hercules, CA, USA) equipped with a CCD camera. The relative expression of proteins with respect to GAPDH was calculated using ImageLab5.2 software.

### 2.5. Histochemical Staining

The tongue and tongue roots taken from the rats were fixed in 10% neutral formalin and then histologically processed and embedded longitudinally in paraffin. Then, 5µ sections were taken on classic slides; hematoxylin-eosin staining was applied all the sections and evaluated under a light microscope.

For histopathological evaluation of the epithelium, the epithelium and cell structure were evaluated, and a 6-grade scoring system was constructed based on the WHO classification of oral epithelial dysplasia reported by Speight [27]. The classification model was described in Table 2. Additionally, hyperkeratinization and vascular dilatation formation were examined using the evaluation method of Ross et al. [28]. The areas where hyperkeratinization and vasodilation were most intense in the sections were determined and these changes were classified into three grades: mild (1), moderate (2), and severe (3).

### 2.6. Statistical Analysis

Gene and protein expressions of all groups were normalized to the mean of the Control groups and data was also normalized with corresponding GAPDH. All data is represented as mean ± standard error of the mean (SEM) through the study. Statistical comparisons were performed using one-way ANOVA followed by appropriate post-hoc test (Tukey). Comparisons giving *p* values less than 0.05 were accepted as statistically significant.

## 3. Results

### 3.1. The Influences of Juglone in the mRNA Expressions of the Apoptosis

**Figure 1:**
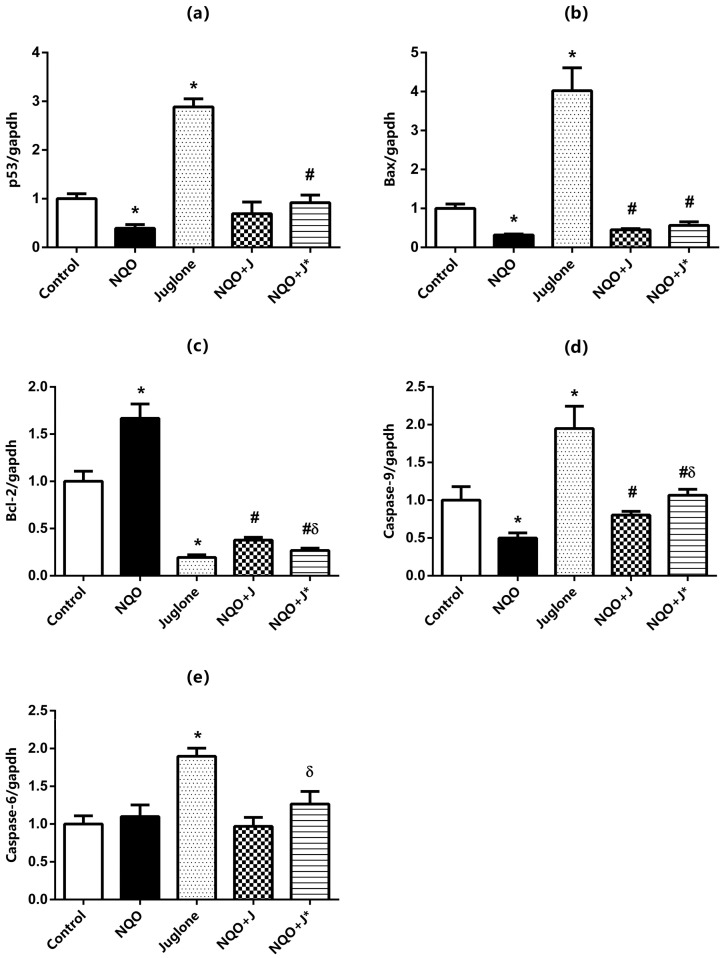
mRNA expression levels of p53 (**a**), Bax (**b**), Bcl-2 (**c**), caspase-9 (**d**), and caspase-6 (**e**) in the tongue of rats from the Control, NQO, Juglone, NQO+J, and NQO+J* groups. Data were normalized by GAPDH. Each bar represents the means of at least six rats. Values are expressed as mean ± SEM. * Significantly different (*p* < 0.05) compared to Control group; # significantly different (*p* < 0.05) compared to NQO group; δ significantly different (*p* < 0.05) compared to NQO+J group.

As shown in Figure 1, the gene expression levels of p53 (a), Bax (b), Bcl-2 (c), caspase-9 (d), and caspase-6 (e) in the tongue tissues from rats were established by real-time PCR analysis. 4-NQO-feeding (NQO group) decreased p53, Bax, and caspase-9 and increased Bcl-2 mRNA expressions in the tongue tissue samples from rats, whereas no changes were observed in caspase-6 mRNA expressions (*p* < 0.05 compared to Control). Intraperitoneal juglone injection (Juglone group) significantly increased p53, Bax, caspase-9, and caspase-6 mRNA expressions in the tongue tissues of healthy rats, whereas markedly reduced Bcl-2 expressions (*p* < 0.05 compared to control). Juglone administration together with NQO-feeding (NQO+J* group) significantly increased p53, Bax, caspase-9, and caspase-6 mRNA expressions and decreased Bcl-2 levels in the tongue tissue samples from rats compared to the NQO groups. Similarly, juglone injection after the carcinogenesis induced by 4-NQO (NQO+J group), Bax and caspase-9 mRNA expressions, while decreased Bcl-2 levels but no changes were observed in p53 and caspase-6 expressions in the tongue tissue samples from rats compared to the NQO groups. On the other hand, caspase-9 mRNA expressions in rats given juglone along with 4-NQO (NQO+J* group) increased significantly more than given juglone after carcinogenesis (NQO+J group). No similar significant change was observed in other parameters.

### 3.2. The Influences of Juglone in the Protein Expressions of the Apoptosis

**Figure 2:**
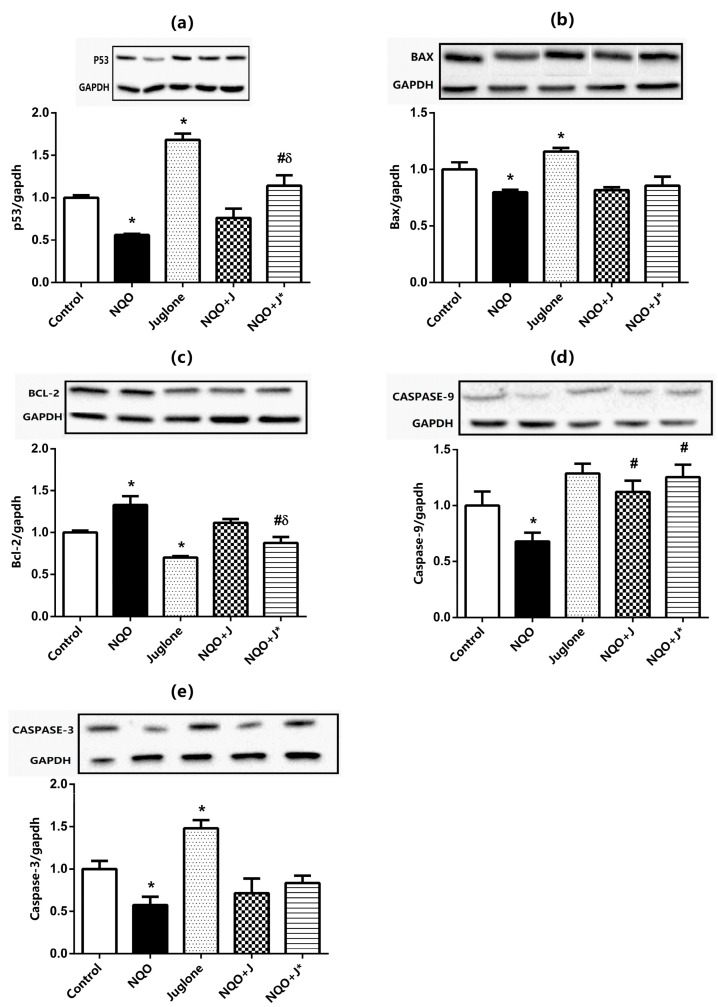
The protein expression levels of insulin receptor substrate p53 (**a**), Bax (**b**), Bcl-2 (**c**), caspase-9 (**d**), and caspase-3 (**e**) in the tongue of rats from the Control, NQO, Juglone, NQO+J, and the NQO+J* groups. The protein levels were quantified using densitometry and normalized with GAPDH. Representative western blot images are included above the corresponding figures. Each bar represents at least six rats. * Significantly different (*p* < 0.05) compared to Control group; # significantly different (*p* < 0.05) compared to NQO group; δ significantly different (*p* < 0.05) compared to NQO+J group.

As shown in Figure 2, the protein expression levels of p53 (a), Bax (b), Bcl-2 (c), caspase-9 (d), and caspase-3 (e) in the tongue tissues from rats were established by *Western blot* analysis. 4-NQO-feeding (NQO group) decreased p53, Bax, caspase-9, and caspase-3 levels and increased Bcl-2 protein expressions in the tongue tissue samples from rats (*p* < 0.05 compared to Control). Intraperitoneal juglone injection (Juglone group) significantly increased p53, Bax, and caspase-3 protein levels in the tongue tissues of healthy rats, and markedly reduced Bcl-2 levels, whereas no changes were found in the caspase-9 expressions (*p* < 0.05 compared to Control). Juglone administration together with NQO-feeding (NQO+J* group) markedly increased p53, and caspase-9 levels and significantly decreased Bcl-2 levels, whereas no changes were observed in the Bax and caspase-3 expressions in the tongue tissue samples from rats compared to the NQO groups. Similarly, juglone injection after the carcinogenesis induced by 4-NQO (NQO+J group), caspase-9 levels were found to be increased compared to NQO group, whereas no changes were observed in the other parameters. On the other hand, rats given juglone along with 4-NQO (NQO+J* group) displayed markedly increased p53 expressions and significantly reduced Bcl-2 levels compared to those given juglone after carcinogenesis (NQO+J group). No similar significant change was observed in other parameters.

### 3.3. The Influences of Juglone on Histological Parameters

**Figure 3:**
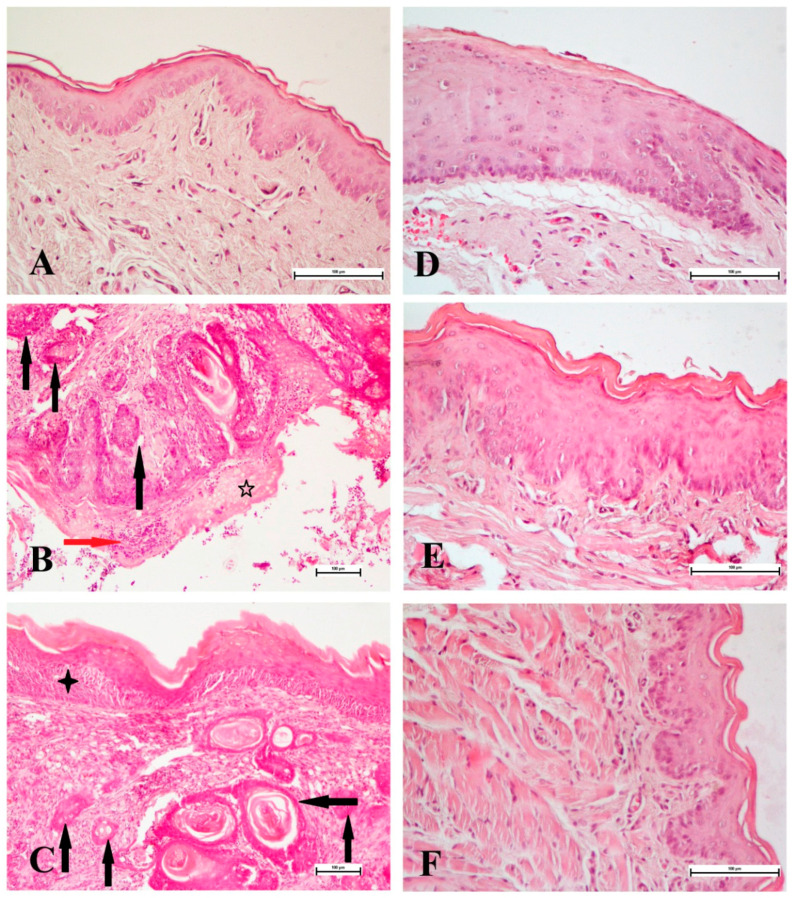
Histopathological features of the tongue tissues from the Control (**A**), NQO (**B**,**C**), Juglone (**D**), NQO+J (**E**), and NQO+J* (**F**) groups. Hematoxylin–eosin staining (**A**,**D**–**F**) ×200, (**B**,**C**) ×100; scale bar: 100 µm. While the Control and Juglone groups showed normal morphologies, epithelial changes and tumor formation are observed in the NQO group (black arrows: tumoral areas; red arrow: lymphocyte infiltration; five-pointed star: hyperkeratinization; four-pointed star: carcinoma in situ). It is observed that epithelial changes are significantly reduced in NQO+J and NQO+J* groups.

As shown in Figure 3, a result of histopathological evaluation under a light microscope, it was observed that the cell arrangements in the Control and Juglone groups were regular and that there was no atypia in their nuclei. However, while hyperkeratotic changes and epithelial thickening were observed in some of the tissues in the Juglone group, mild dysplasia was observed in only one sample. Again, hyperkeratinization and vascular dilatation were observed to be mild in these groups. While moderate dysplasia was observed extensively in the NQO group, carcinoma was observed in situ to develop in one sample. Again, severe hyperkeratinization and severe vascular dilatation were observed in this group. While hyperplasia was observed in most of the samples in the NQO+J group, carcinoma was observed in situ in one sample. While mild hyperkeratinization was observed in this group, moderate vascular dilatation was observed. In the NQO+J* group, while most of the samples showed hyperplasia, mild dysplasia was observed in one sample. Hyperkeratinization and vascular dilatation were observed to a mild degree (Table 3).

## 4. Discussion

Oral cancer consists of cancer of areas of the oral cavity including, the lips, labial and buccal mucosa, anterior two-thirds of the tongue, maxillary and mandibular gums, retromolar region, floor of the mouth under the tongue, and roof of the mouth. These areas are histologically covered with squamous epithelium (keratinized/non-keratinized, masticatory, special mucosa). The remainder consists of oral cancer, verrucous carcinoma, minor salivary gland malignancies, mucosal melanoma, Kaposi’s sarcoma, primary intraosseous squamous cell carcinoma, osteosarcoma, odontogenic tumors, metastatic tumors, and connective tissue tumors [29]. One of the basic features that the cell often, but not always, acquires is the ability to escape from apoptosis. Apoptosis, technically called programmed cell death, is an important element in the pathogenesis of many diseases [30]. It is known that the initiation of apoptosis occurs via trans-membrane receptor-mediated interactions or caspase-initiated mitochondria. According to Jorge Finnigan, TNF (ligands and receptors), p53 family, Bax/Bcl-2 family, as well as caspases, are noteworthy among the genes involved in apoptosis [31].

Recent studies have focused on the role of PIN1 inhibition in this process. In human breast cancer, PIN1 promotes oncogenesis via the cyclin D1 regulation. Studies have shown that PIN1 increases cyclin D1 transcription in association with the HER2–HRAS–JNK–AP1, WNT–β-catenin, and NF-κB pathways. PIN1 regulates HER2, NOTCH1, NOTCH3, androgen receptor (AR) and estrogen receptor α (ERα), which are cancer-driving receptors Furthermore, PIN1 regulates AMPK, AKT93, MYC, PKM2, RAF1, SMAD2, SMAD3, STAT3, the RAS family member RAB2A28, FAK, protein tyrosine phosphatase, PTP-PEST, S6K, and SGK1, which act as intracellular signaling modulators. PIN1 induces the interaction of non-receptor type 12 (PTP-PEST) with FAK to increase the FAK Tyr397 dephosphorylation, which induces cancer metastasis. PIN1 also promotes epithelial–mesenchymal transition (EMT) of MCF-7 cells by inducing the transcriptional activity of STAT3 and recruiting its transcription coactivator p300. PIN1 induces cancer metastasis and invasion by activating β-catenin, BRD4, NF-κB, and p53M. Overexpression of PIN1 increases the PTOV1 expression as a novel interactome of PIN1, and knockdown of both genes inhibits the expression of β-catenin, cyclin D1, and c-Myc in breast cancer MDA-MB-231 cells [12].

PIN1 is a member of a group of three prolyl isomerases. PIN1 interacts with the motif containing the phospho-Ser/Thr-Pro of substrates and increases the cis–trans isomerization of peptide bonds, thereby controlling the functions of these substrates. Importantly, PIN1 expression level is highly upregulated in most cancer cells and is associated with malignant features and therefore poor outcomes. Additionally, PIN1 was revealed to promote the functions of multiple oncogenes and eliminate tumor suppressors. Accordingly, PIN1 is well known as a key regulator of malignant processes [32]. It is clearly stated that PIN1 activation has an anti-apoptotic effect [33]. It has also been shown to have a role in the regulation of caspases [34]. Hyperactivated PIN1 has been shown to cause nasopharyngeal carcinoma [35]. A study conducted in Eastern China found that the risk of oral squamous cell carcinoma was significantly increased in 209 patients with PIN1 polymorphism [36]. Therefore, it can be said that juglone, a strong PIN1 inhibitor, may be a potential candidate for treatment in oral cancer models. In this study, an oral carcinoma model was tried to be created with 4-NQO, and some parameters in the apoptotic pathway were measured by administering juglone both during this process and after 4-NQO application. According to the study results, it was determined that the apoptotic factors p53, Bax, and caspase-9 mRNA and protein expressions, as well as caspase-3 enzyme levels, were suppressed with 4-NQO, while the anti-apoptotic Bcl-2 mRNA and protein expressions were increased. In addition, histological examinations showed intense dysplasia with 4-NQO, hyperkeratinization, and severe vascular dilatation in one sample and carcinoma in situ. These results indicate that an oral carcinoma model was formed with 4-NQO, in line with the literature [37]. It has been shown that juglone administration causes Bax hyperactivation in the Ehrlich carcinoma model developed on male BALB/c mice [38]. In a similar study, it was shown that juglone fractions caused the induction of p53, caspase-9, caspase-3, and Bax while suppressing Bcl-2 levels [17]. In the nasopharyngeal carcinoma model created by PIN1 induction, juglone was shown to increase caspase-3 protein expression [39]. In our study, juglone was applied for prophylactic and therapeutic purposes at a dose compatible with the literature (1 mg/kg) [40]. Study results indicate that Bax and caspase-9 mRNA expressions from apoptotic structures increased by administering juglone to healthy rats together with 4-NQO and to rats with oral carcinoma induced by 4-NQO, while p53 levels increased significantly in the 4-NQO+J* group, and there is an increasing trend in the 4-NQO+J group. Additionally, juglone was found to dramatically suppress Bcl-2 mRNA expression in all groups. When the protein expressions are evaluated, it is understood that the results are largely parallel to mRNA expressions, and as a different factor, caspase-3 values are also induced by juglone application. It can be said that our results are compatible with the effects of juglone in other cancer models. Another point worth noting here is that the anti-carcinogenic effects of juglone are more pronounced at the prophylactic level (when given together with 4-NQO). This may be particularly related to the regulatory activity of PIN1 on apoptotic/anti-apoptotic structures. The fact that the effects of juglone are more aggressive at the scope of the mRNAs in healthy rats raises questions about the safety of juglone. In a study on the subject, it was stated that the IC50 values of juglone can kill prokaryotic cells without damaging eukaryotic cells. Thus, juglone can be used in pharmaceutical fungicidal and bactericidal applications as well as food safety applications and agriculture [41]. In our study, we can say that although juglone’s apoptosis was clearly expressed at the based on the mRNAs, this was not reflected in protein synthesis to the same extent. This may be related to the fact that apoptosis is regulated through different pathways.

While dysplasia develops histologically in oral carcinoma, hyperkeratinization, severe vascular dilatation, and even carcinoma are likely to be observed in situ [37]. In our study, the histological findings obtained by examinations under a light microscope were evaluated with Speights’ scoring [27]. The results showed that low-level hyperkeratotic changes and epithelial thickening occurred with juglone application in healthy rats. It was determined that severe hyperkeratinization, high dysplasia and vascular dilatation that developed with 4-NQO were alleviated in both groups administered juglone. In parallel with the molecular results, these changes were found to be more pronounced in rats given juglone together with 4-NQO.

Current data suggest that the possible effects of juglone may be mediated by regulation of the apoptosis signaling pathway due to PIN1 inhibition. Although there are limited studies on the effects of juglone, our findings were found to be compatible with juglone’s pancreatic, prostate, melanoma, colorectal, glioblastoma, breast, Ehrlich ascites, and nasolaryngeal cancer models [17,19,21,22,23,24]. Further studies are needed to substantiate our hypothetical assessments.

## 5. Conclusions

Oral carcinoma has emerged as one of the most prevalent forms of cancer in recent times. Juglone is a naturally occurring agent that mediates PIN1 inhibition, offering a novel approach to cancer treatment. To date, no studies have examined the effects of juglone on oral carcinoma. These findings demonstrate that juglone effectively reduces the development of carcinoma in the tongue and exhibits this activity in both treatment and prophylactic use. The results of further in vivo studies investigating the effects of juglone may lead to a change in the perspectives of clinicians regarding treatment and inspire future studies on juglone.

## Figures and Tables

**Table 1:** Primer sequences of p53, Bax, Bcl-2, Caspase-9, Caspase-6, and the internal standard GAPDH used for the mRNA expression determination of qRT-PCR.

Gene	Forward Primer Sequence (5′ → 3′)	Reverse Primer Sequence (3′ → 5′)
p53	TCCCCTGAAGACTGGATAACT	TTCCTCTGGGCCTTCTAACA
Bax	GCTCAGCTTCTTGGTGGATG	CCTTTTTGCTACAGGGTTTCAT
Bcl-2	TTCCTGCATCTCATGCCAAG	TACCAATAGCACTTCGCGTC
Caspase-9	ATGGTCTTTCTGCTCACCAC	GTCACGGCTTTGATGGAGAT
Caspase-6	GACCGACTAAAACAGGCCC	AATTACTGTGCGCAAATGCC
GAPDH	TGATGACATCAAGAAGGTGGTGAAG	TCCTTGGAGGCCATGTGGGCCAT

**Table 2:** Morphological changes according to Speights’ scores.

Morphology	Score	Cell Structure	Epithelial Structure
Normal epithelium	0	No change	No change
Hyperplasia	1	No change	Although normal maturation, epithelial thickening and hyperkeratosis
Mild dysplasia	2	Differentiation in cell and nucleus shapes in the lower 1/3 of the epithelium	Thickening in the lower 1/3 of the epithelium, disruption of cell orientations
Moderate dysplasia	3	Differentiation in cell and nucleus shapes up to the middle 1/3 of the epithelium	Increase in the number of cells up to the middle 1/3 of the epithelium, thickening of the epithelium, complete disruption of cell orientation
Severe dysplasia	4	Disruption of the full-thickness cell structure of the epithelium	Migration of atypical cells from the basal lamina to the lamina propria in addition to the disrupted epithelial structure
Carcinoma in situ	5	All changes available	Full-layer structural changes and delamination

**Table 3:** Histopathological changes of the tongue tissues from the Control, NQO, Juglone, NQO+J, and the NQO+J* groups.

Groups	Oral Epithelial Dysplasia	Hyperkeratinization	Vascular Dilatation
Control	0	1	1
NQO	3.57 ± 0.57	2.83 ± 0.17	2.67 ± 0.21
Juglone	0.84 ± 0.31	1.16 ± 0.17	1
NQO+J	1.33 ± 0.21	1	1.5 ± 0.22
NQO+J*	1.67 ± 0.67	1.5 ± 0.34	1.33 ± 0.33

## Data Availability

The data that support the findings of this study are available from the corresponding author upon reasonable request.
